# Identification of Diagnostic Biomarkers for Colorectal Polyps Based on Noninvasive Urinary Metabolite Screening and Construction of a Nomogram

**DOI:** 10.1002/cam4.70762

**Published:** 2025-04-08

**Authors:** Yang Xie, Yiyi Jin, Zide Liu, Jun Li, Qing Tao, Yonghui Wu, Youxiang Chen, Chunyan Zeng

**Affiliations:** ^1^ Department of Gastroenterology Jiangxi Province Hospital of Integrated Chinese and Western Medicine Nanchang Jiangxi China; ^2^ Department of Gastroenterology, Jiangxi Provincial Key Laboratory of Digestive Diseases, Jiangxi Clinical Research Center for Gastroenterology, Digestive Disease Hospital, the First Affiliated Hospital, Jiangxi Medical College Nanchang University Nanchang Jiangxi China

**Keywords:** colorectal polyp, machine learning, nomogram, urine metabolomics

## Abstract

**Purpose/Backgrounds:**

Colorectal polyps (CRPs) are precursors to colorectal cancer (CRC), and early detection is crucial for prevention. Traditional diagnostic methods are invasive, prompting a need for noninvasive biomarkers. This study aimed to identify urinary metabolite biomarkers for diagnosing CRPs and construct a diagnostic nomogram based on noninvasive urinary metabolite screening.

**Patients and Methods:**

A total of 192 participants, including 64 CRP patients and 128 healthy controls, were recruited. Urine samples were analyzed using untargeted gas chromatography–mass spectrometry (GC–MS) and ultra‐performance liquid chromatography–mass spectrometry (UPLC–MS). Metabolite screening was performed using weighted gene coexpression network analysis (WGCNA), least absolute shrinkage and selection operator (LASSO), and support vector machine‐recursive feature elimination (SVM‐RFE). A diagnostic nomogram was developed based on identified metabolites, and its performance was evaluated using receiver operating characteristic (ROC) curves, calibration plots, and decision curve analysis (DCA).

**Results:**

A total of 350 metabolites were identified, with 7 key metabolites significantly associated with CRP. Multivariate logistic regression analysis identified Saccharin (OR = 48.3, 95% CI: 4.44–525.32) and N‐omega‐acetylhistamine (OR = 27.91, 95% CI: 2.31–337.06) as significant risk factors for CRP, while N‐methyl‐L‐proline, trimethylsilyl ester (OR = 0.08, 95% CI: 0.01–0.8) was a protective factor. A nomogram incorporating these metabolites demonstrated strong discriminatory power, with AUC values of 0.974 and 0.960 in the training and validation sets, respectively. Calibration plots and DCA confirmed the model's accuracy and clinical utility.

**Conclusions:**

This study successfully identified seven urinary metabolites as potential noninvasive biomarkers for CRP. The constructed diagnostic nomogram, based on these metabolites, offers high predictive accuracy and clinical applicability, providing a promising tool for the early detection of CRP.

## 1 Introduction

Colorectal cancer (CRC) is the third most diagnosed cancer worldwide, with over 1.9 million new cases and 935,000 deaths reported in 2020 alone [1]. The global burden of CRC is expected to rise, particularly in developing countries, due to aging populations and lifestyle changes [2]. Early detection and removal of colorectal polyps (CRPs), the precursors to CRC, are critical for preventing the progression to CRC, significantly improving patient outcomes [3]. Currently, colonoscopy is the gold standard for the detection of CRPs, but its invasiveness, cost, and requirement for bowel preparation limit its widespread application in population‐based screening programs. Therefore, there is a pressing need for noninvasive, cost‐effective, and reliable diagnostic methods to identify individuals at high risk for CRPs.

Several risk factors contribute to the development of CRPs and CRC, including age, sex, family history, and lifestyle factors such as diet, smoking, and alcohol consumption [4, 5]. In addition, chronic stress and widespread use of antibiotics that alter the gut microbiota may also promote the early onset of CRC [6]. We know that patients with inflammatory bowel disease [7], hereditary nonpolyposis CRC [8], familial adenomatous polyposis [9], and metabolic syndrome [10] are at increased risk for CRC. A recent prospective study from the United States found that patients with type 2 diabetes have a significantly increased risk of CRC [11]. A Mendelian randomization study found that higher fasting insulin levels increased the risk of CRC [12]. Rossella et al.'s study has also highlighted the role of chronic kidney disease (CKD) in increasing the risk of CRP and CRC [13]. Patients with CKD exhibit elevated levels of uremic toxins, which have proinflammatory and carcinogenic effects, potentially promoting the development of CRPs. Therefore, the search for CRP biotargets along with the reduction of these risk factors is particularly important for early monitoring of CRC progression. Recent advances in metabolomics have opened new avenues for the identification of disease‐specific biomarkers [14, 15]. Urine, in particular, has emerged as a valuable biofluid for metabolomic studies due to its noninvasive collection, ease of handling, and the comprehensive metabolic information it can provide [16]. Urinary metabolomics has shown promise in identifying biomarkers for various diseases, including cancers, by reflecting the biochemical alterations associated with disease states [17]. However, research focusing on urinary metabolomics for the early detection of CRP remains limited.

In this study, we employed advanced metabolomic techniques, including gas chromatography–mass spectrometry (GC–MS) and ultra‐performance liquid chromatography–mass spectrometry (UPLC–MS), to identify urinary biomarkers for CRP. These techniques provide high sensitivity, specificity, and the ability to detect a wide range of metabolites, making them superior to traditional methods for biomarker discovery. GC–MS is particularly effective for volatile and thermally stable compounds, while UPLC–MS offers high‐resolution and rapid analysis of complex biological samples [18]. By combining these techniques, we were able to comprehensively profile the urinary metabolome and identify key metabolites associated with CRP.

Nomogram is a reliable predictive model that quantifies the risk of clinical events through a simple and intuitive chart [19]. There are many previous studies on diagnostic nomograms constructed for CRC [20]. There are no nomograms developed for people at high risk of CRPs. In this study, we aim to identify potential diagnostic biomarkers for CRPs through the screening of urinary metabolites. By employing advanced metabolomic techniques, we will analyze the metabolic profiles of patients with CRPs and healthy controls to identify significant differences. Furthermore, we will construct a nomogram based on these biomarkers to develop a noninvasive diagnostic tool for the early detection of CRPs. The integration of noninvasive biomarkers into large‐scale screening programs has the potential to significantly reduce the burden on healthcare systems. Traditional screening methods, such as colonoscopy, are resource‐intensive and require specialized equipment and trained personnel. In contrast, urinary metabolomic profiling offers a cost‐effective, noninvasive alternative that can be easily implemented in routine clinical practice. By identifying individuals at high risk for CRP through simple urine tests, healthcare providers can prioritize high‐risk patients for further diagnostic evaluation, thereby optimizing resource allocation and improving early detection rates [8]. The development of a diagnostic nomogram based on urinary metabolites, as presented in this study, represents a significant step toward the implementation of noninvasive screening tools in clinical practice. This approach has the potential to facilitate early intervention, reduce the incidence of CRC, and improve overall patient outcomes.

## 2 Materials and Methods

### 2.1 Participating Subjects

This study included 192 participants, comprising 64 patients with CRPs and 128 healthy controls. All samples were randomly divided into a training set and a validation set in a 7:3 ratio. Urine samples were collected from patients treated at the First Affiliated Hospital of Nanchang University between March and December 2019. The study was conducted in accordance with the Declaration of Helsinki and was approved by the Clinical Research Ethics Committee of the First Affiliated Hospital of Nanchang University. Informed consent was obtained from all participants.

The inclusion criteria for the study subjects were as follows: (1) The healthy control group consisted of individuals who had undergone medical examinations and had no findings of CRP on endoscopic examination; (2) Patients were diagnosed with CRP based on endoscopic biopsy pathology; and (3) Participants provided comprehensive clinical information. Exclusion criteria included: (1) Patients with malignancies or other infectious diseases; (2) Patients with severe metabolic diseases or a long‐term history of medication; (3) Patients with acute conditions requiring emergency treatment; and (4) Patients who had experienced major illnesses or undergone significant surgeries in the past 3 months, such as malignancies, severe cardiovascular diseases, major organ transplants, or conditions leading to lifelong disability, advanced chronic diseases, deep coma, permanent paralysis, or severe brain injury. Clinical characteristics were collected from the hospital's electronic medical record system, including demographic features (age and sex). The outcome variable was the diagnosis of CRP.

### 2.2 Sample Preprocessing

Urine samples were collected from both patients and healthy controls and processed as previously described [21, 22]. In brief, 5 mL of urine was collected and centrifuged at 3800 rpm for 10 min at 4°C to obtain the supernatant. For sample preparation, 50 μL of the urine was taken, and 700 μL of MTBE buffer containing internal standards—0.45 μg/mL gibberellic acid A3, 1 μg/mL 13C sorbitol, and 0.45 μg/mL PC (17:0/14:1)—was added. After mixing with an inverter, 350 μL of methanol/water (1:3, v/v) was added to the mixture, leading to phase separation. The lipophilic phase and hydrophilic phase were then separately collected for lipid and metabolite analysis.

### 2.3 Untargeted LC–MS/MS Analysis and Metabolite Identification

In our study, metabolites were detected and identified using gas chromatography–mass spectrometry (GC–MS) and ultra‐performance liquid chromatography–mass spectrometry (UPLC–MS), following the methods previously described [21]. For GC–MS analysis, urine samples were first derivatized to enhance the volatility and stability of metabolites. Briefly, 50 μL of urine supernatant was mixed with 20 μL of methoxyamine hydrochloride and incubated at 37°C for 90 min to protect carbonyl groups. Subsequently, 80 μL of N‐methyl‐N‐(trimethylsilyl) trifluoroacetamide (MSTFA) was added, and the mixture was incubated at 37°C for 30 min to derivatize hydroxyl and amine groups. The derivatized samples were then analyzed using a GC system coupled with a mass spectrometer. The GC separation was performed on a DB‐5MS capillary column, with helium as the carrier gas at a flow rate of 1.0 mL/min. Metabolites were identified by comparing their mass spectra with those in the NIST 2017 library and by matching their retention indices with those of known standards. For UPLC–MS analysis, urine samples were analyzed using a Waters ACQUITY UPLC system coupled with a Xevo G2‐XS Q‐TOF mass spectrometer. Metabolites were separated on an ACQUITY UPLC HSS T3 column maintained at 40°C. The mobile phase consisted of (A) water with 0.1% formic acid and (B) acetonitrile with 0.1% formic acid. The flow rate was 0.4 mL/min, and the injection volume was 2 μL. The capillary voltage was set to 2.5 kV, and the source temperature was 120°C. The desolation gas flow was 800 L/h at a temperature of 450°C. Metabolites were identified by matching their accurate masses and retention times with those in the Human Metabolome Database (HMDB) and Kyoto Encyclopedia of Genes and Genomes (KEGG) databases. To ensure the reliability of our analysis, we applied a frequency threshold of 50%, meaning that only metabolites detected in at least 50% of the samples in either the CRP group or the healthy control group were included in the statistical analysis. This threshold was chosen to minimize the impact of sporadic or rare metabolites on the results and to focus on metabolites that were consistently present across the study population.

### 2.4 Weighted Gene Coexpression Network Analysis (WGCNA)

WGCNA is not confined to gene expression data; it can also be applied to metabolomics data to identify coexpression modules of metabolites and explore their associations with specific traits or conditions [23, 24]. WGCNA was chosen for its ability to identify coexpression modules of metabolites and explore their associations with specific traits or conditions. This approach is particularly useful for uncovering metabolic pathways and processes underlying disease phenotypes [24]. When used in metabolomics, WGCNA helps categorize metabolites into modules based on their correlation patterns, offering insights into the metabolic pathways and processes underlying specific phenotypes or diseases [24]. The steps involved in applying WGCNA to metabolomics are similar to its use in gene expression data: first, metabolite data are preprocessed, including normalization and outlier detection, to ensure reliable results. Next, a network is constructed by calculating pairwise correlations between metabolites and creating an adjacency matrix that represents the weighted network. Modules are then identified using hierarchical clustering to group coexpressed metabolites, which often correspond to specific metabolic pathways or related biochemical processes. Finally, module–trait associations are analyzed to link the identified metabolite modules to clinical traits, determining which key metabolites are significantly related to the phenotype. The soft‐thresholding power was set to 9, and dynamic module detection was performed to group metabolites into modules based on their correlation patterns. This approach is particularly useful in metabolomics studies aimed at identifying biomarkers or understanding metabolic changes associated with diseases.

### 2.5 Least Absolute Shrinkage and Selection Operator (LASSO)

LASSO regression was employed for its ability to perform both feature selection and regularization, effectively identifying key metabolites associated with CRP while reducing model complexity [25]. Lasso regression is a type of linear regression that includes a penalty term to constrain the coefficients of the model. The penalty term, which is the sum of the absolute values of the coefficients multiplied by a tuning parameter (λ), helps to perform both regularization and feature selection [26]. By applying this penalty, LASSO regression was applied to select key metabolites by shrinking the coefficients of less important variables to zero. This results in a model that only includes a subset of the original features, making it particularly useful for identifying key metabolites associated with a given outcome [27].

### 2.6 Support Vector Machine‐Recursive Feature Elimination (SVM‐RFE)

SVM‐RFE is a feature selection technique based on the support vector machine (SVM) classifier [28]. It works by recursively eliminating features and evaluating the model's performance at each iteration [29]. SVM‐RFE was selected for its robustness in ranking features based on their importance and iteratively eliminating the least significant ones, ensuring the identification of the most relevant biomarkers. In each step, SVM‐RFE ranks features according to their importance, such as their weight in the SVM model, and removes the least important ones. This process continues until the desired number of features is reached [29]. The method involves training the SVM model with all features, ranking the features based on their contribution to the model's performance, and then eliminating the least important features while retraining the model, iterating this process to identify the optimal feature set. SVM‐RFE was used to recursively eliminate the least important features based on their contribution to the model's performance. The intersection of metabolites identified by WGCNA, LASSO, and SVM‐RFE was used to select the final set of biomarkers for further analysis.

### 2.7 Nomogram Construction Process

Participants were divided into two groups based on clinical evaluation results: healthy individuals and patients with CRPs. Metabolite results were converted into binary variables according to the optimal cut‐off values derived from ROC analysis [30]. The cut‐off values for the metabolites were as follows: PP_0017942: 28.13; GC1263: 43.42; GC1457: 42.64; GC1484: 35.25; PN_0023236: 11.71; PN_0024153: 46.64; and PN_0039851: 31.98. The construction of the diagnostic nomogram for predicting CRP patients was carried out in three steps: (1) Metabolites were used as binary categorical variables for logistic regression analysis in the training set; (2) A multivariable logistic regression model was fitted using variables with *p* < 0.05 from the univariable logistic regression analysis; (3) The nomogram was constructed based on the results of the multivariable logistic regression. Additionally, the predictive performance of the nomogram in terms of discrimination, calibration, and clinical utility was validated using 1000 bootstrap resampling iterations. The area under the ROC curve (AUC) was used to evaluate the model's ability to distinguish between healthy individuals and CRP patients. Calibration was assessed using the Hosmer–Lemeshow test and by comparing the predicted and observed probabilities of CRP diagnosis. Decision curve analysis (DCA) was performed to evaluate the clinical utility of the predictive nomogram.

### 2.8 Statistical Analysis

The enrolled patient cohort was randomly divided into a development group and a validation group. To ensure that both the development and validation groups had sufficient sample sizes, thereby guaranteeing the stability and reliability of the model, we ultimately chose a 7:3 ratio for the split. Categorical data are presented as frequencies and percentages. Continuous variables were analyzed using independent *t*‐tests or Mann–Whitney U tests, depending on the data distribution, while categorical data were analyzed using chi‐square tests or Fisher's exact tests. Logistic regression analysis was used to identify risk factors associated with the potential for CRP, and the results are expressed as odds ratios (OR) with 95% confidence intervals (CI). Multivariate logistic regression analysis was performed to construct a nomogram model for predicting the risk of CRP. The performance of the model was validated using the validation group, and its accuracy was assessed using the Hosmer–Lemeshow test. The discriminative ability of the predictive model was evaluated by calculating the area under the receiver operating characteristic (ROC) curve (AUC) and determining the optimal cut‐off value. Calibration curves were used to assess the predictive consistency of the model, and decision curve analysis (DCA) was performed to evaluate its clinical utility. All statistical analyses were conducted using R version 4.3.3 (https://www.r‐project.org) or GraphPad prism 8.0. A significance level of *p* < 0.05 was considered statistically significant.

## 3 Results

### 3.1 Analysis Process of Metabolites

**FIGURE 1:**
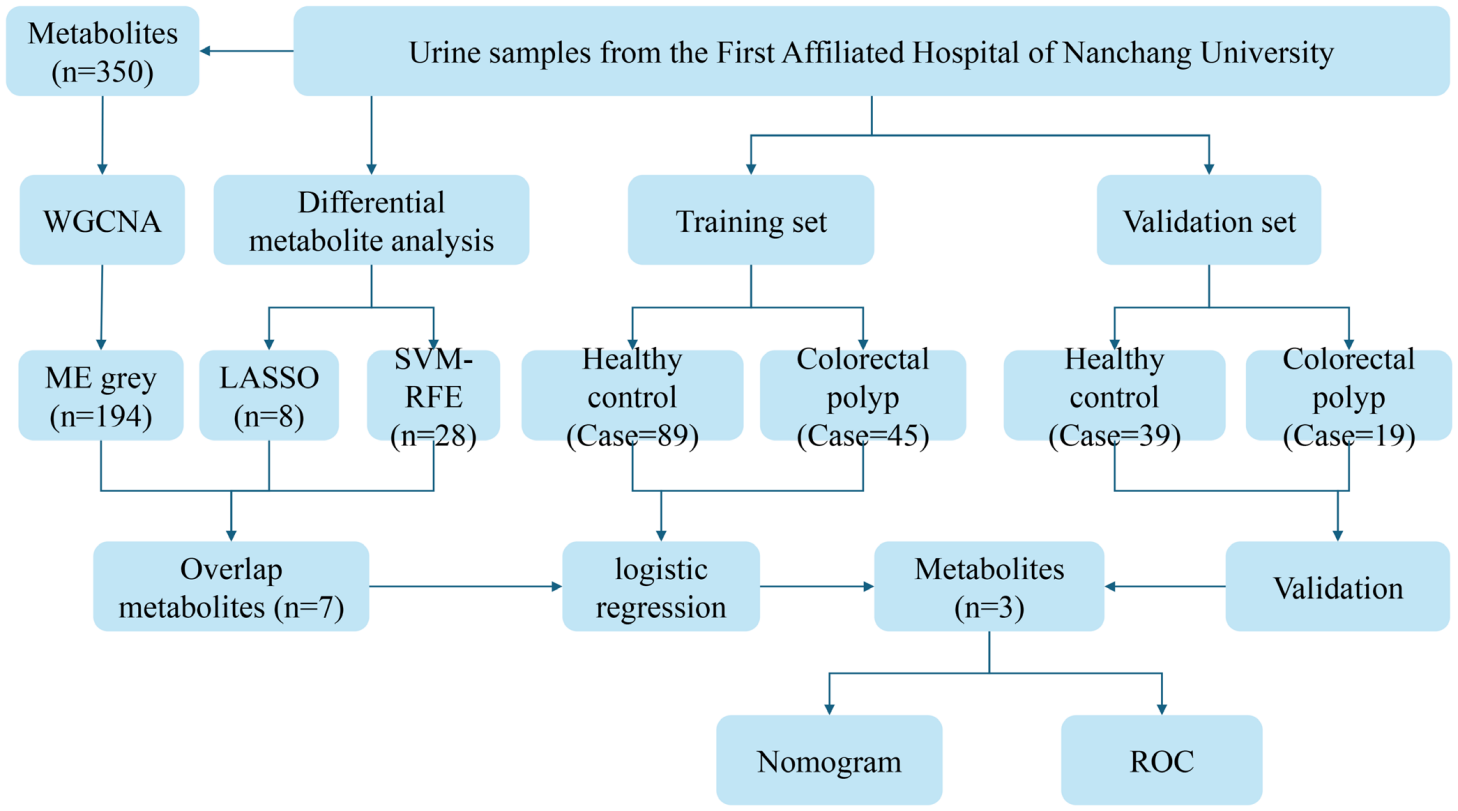
Flow chart of the study. The “*n*” represents the number of metabolites.

A total of 350 compounds were identified, with 200 identified by GC–MS and 150 by UPLC–MS. These metabolites were classified into various categories, including lipids, organic acids, amino acids, peptides, fatty acids, nucleotides, carbohydrates, bile acids, the TCA cycle, and xenobiotics, based on the Kyoto Encyclopedia of Genes and Genomes (KEGG) and HMDB databases. Figure 1 is a flow chart of the design of this study.

### 3.2 Screening Metabolites by Machine Learning in all Samples

**FIGURE 2:**
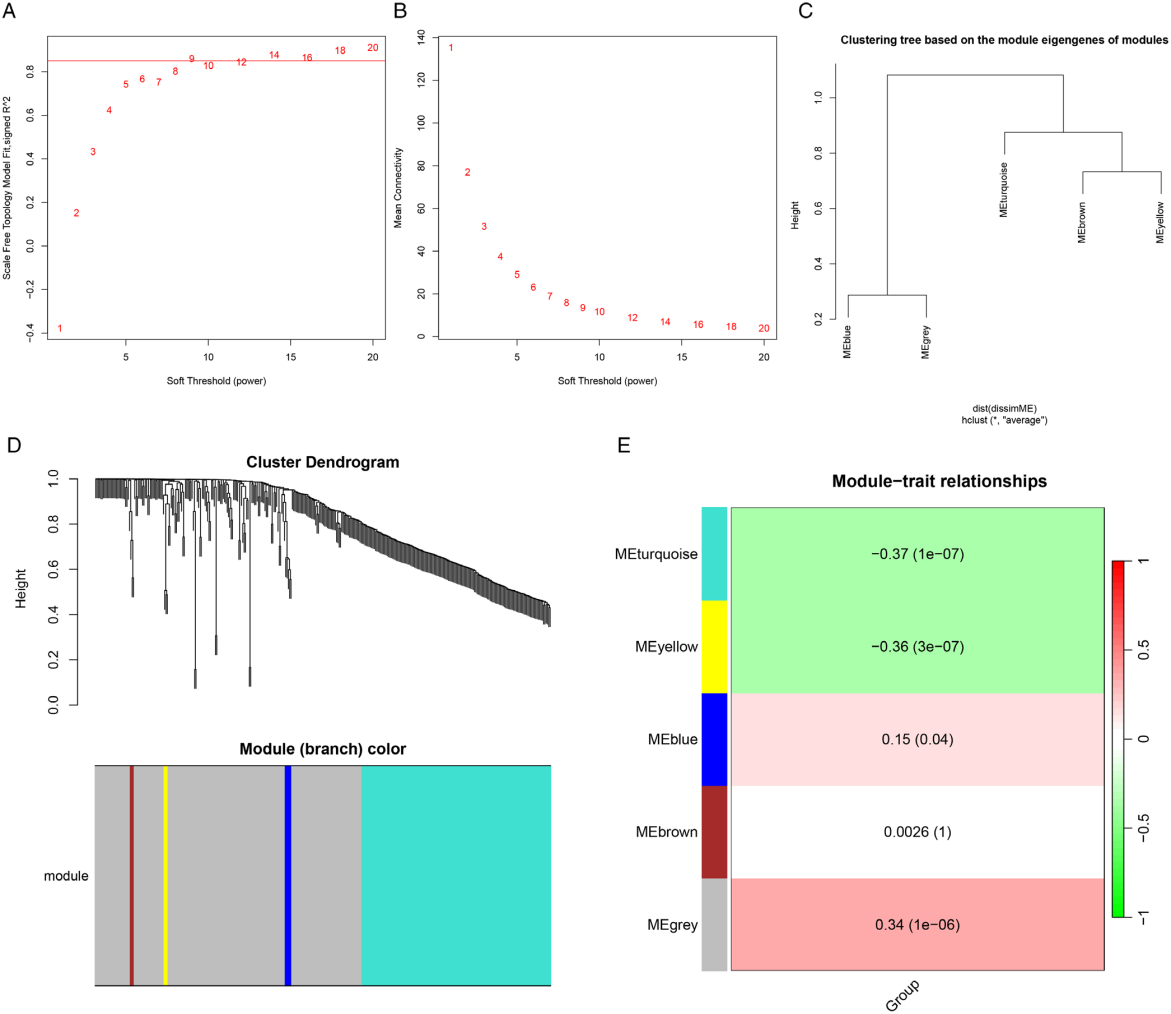
Screening process in patients with CRPs and healthy controls by WGCNA algorithm. (A) Scale independence and (B) mean connectivity. (C) Clustering tree based on the module eigengenes of modules. (D) Gene dendrogram and module colors. (E) Pearson correlation analysis of merged modules.

**FIGURE 3:**
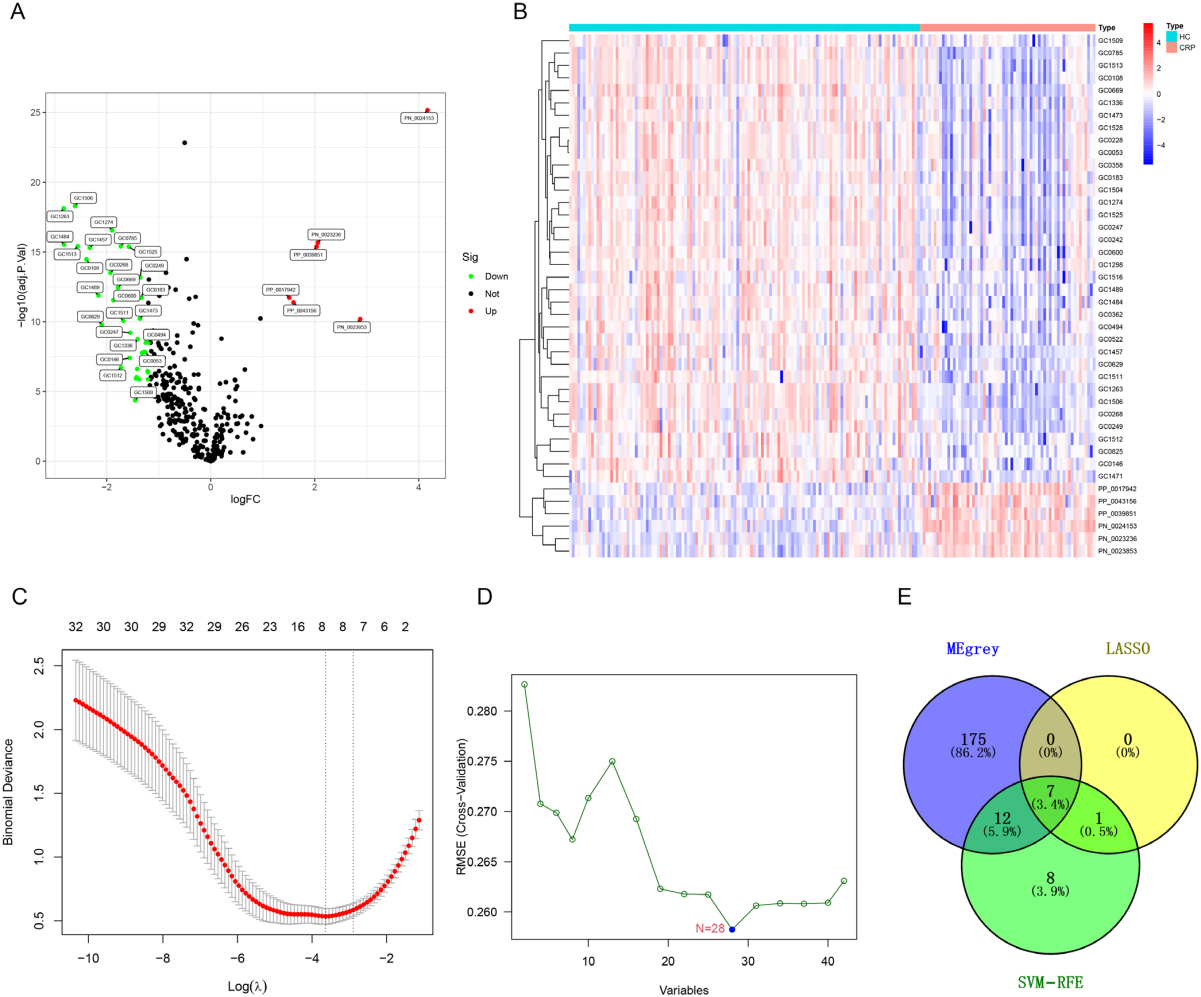
Screening process in patients with CRPs and healthy controls by differential analysis (A–B), lasso (C), and SVM‐RFE algorithm (D). (E) Overlapped metabolites.

**FIGURE 4:**
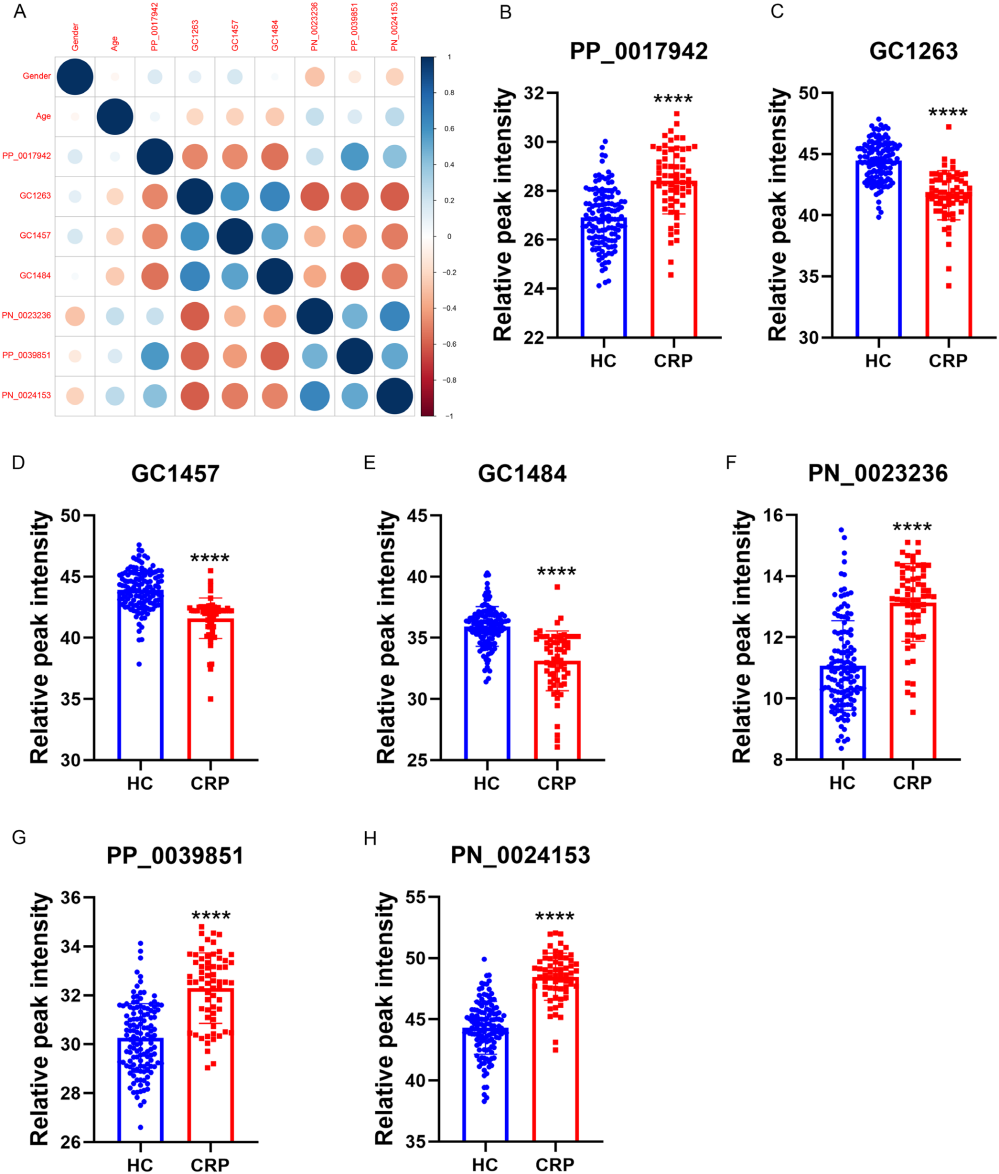
Correlation analysis and relative peak intensity of metabolites in all samples. (A) Correlation analysis of seven metabolites, age, and sex. (B) PP_0017942, 3‐(1H‐Indol‐3‐yl)‐2‐(trimethylammonio) propanoate; (C) GC1263, 3‐(3‐hydroxyphenyl)‐3‐hydroxypropionic acid; (D) GC1457, N‐methyl‐L‐proline, trimethylsilyl ester; (E) GC1484, 3,4‐dihydroxyhydrocinnamic acid NIST; (F) PN_0023236, hippuric acid; (G) PP_0039851, N‐omega‐acetylhistamine; (H) PN_0024153, saccharin. ****p < 0.0001.

**FIGURE 5:**
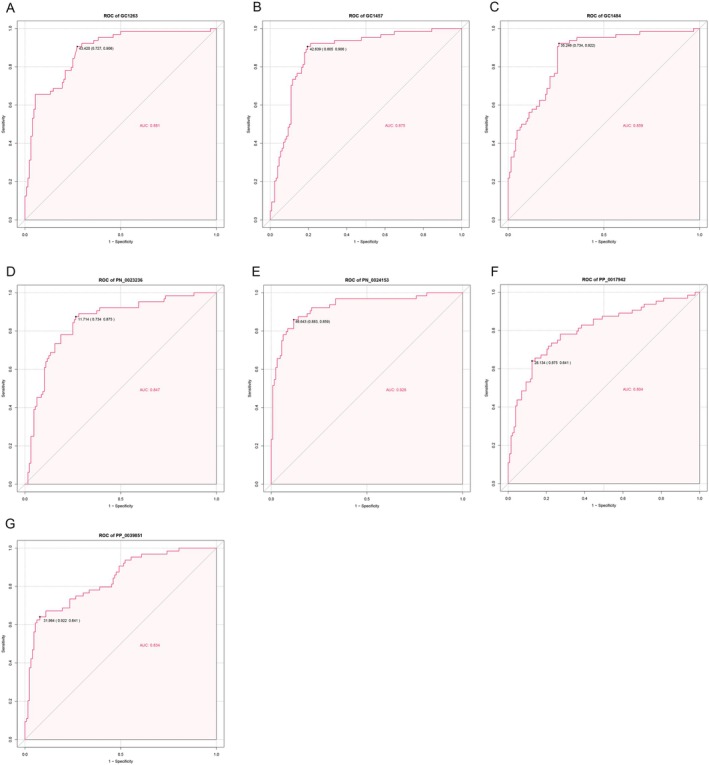
Diagnostic value of seven metabolites in all samples. (A) GC1263, 3‐(3‐hydroxyphenyl)‐3‐hydroxypropionic acid; (B) GC1457, N‐methyl‐L‐proline, trimethylsilyl ester; (C) GC1484, 3,4‐dihydroxyhydrocinnamic acid NIST; (D) PN_0023236, hippuric acid; (E) PN_0024153, saccharin; (F) PP_0017942, 3‐(1H‐indol‐3‐yl)‐2‐(trimethylammonio) propanoate; (G) PP_0039851, N‐omega‐acetylhistamine.

In this study, we selected 350 compounds for WGCNA, with a soft‐thresholding power of 9 (Figure 2A). Dynamic module detection was then performed, requiring a minimum of three metabolites per module (Figure 2B,C). A total of five coexpression modules were identified, among which the gray module (Correlation = 0.34, *p* = 1e‐06) exhibited the strongest positive correlation with CRPs (Figure 2D). From the gray module, 194 metabolites were identified as potential CRP‐related candidates. Additionally, we performed differential analysis on the 350 annotated metabolites, identifying 36 metabolites that were significantly decreased in the urine of CRP patients and 6 metabolites that were significantly increased in the urine of CRP patients compared to healthy controls. The corresponding volcano plot and heatmap are shown in Figure 3A,B, respectively. Using LASSO and SVM‐RFE methods, 8 and 28 differential metabolites were identified, respectively (Figure 3C,D). By intersecting the results of WGCNA, LASSO, and SVM‐RFE analyses, we identified seven common metabolites (Figure 3E), which include: 3‐(1H‐indol‐3‐yl)‐2‐(trimethylammonio) propanoate; 3‐(3‐hydroxyphenyl)‐3‐hydroxypropionic acid; N‐methyl‐L‐proline, trimethylsilyl ester; 3,4‐dihydroxyhydrocinnamic acid NIST; hippuric acid; N‐omega‐acetylhistamine; and saccharin. We performed correlation analysis between these metabolites and clinical factors such as sex and age, with the correlation heatmap presented in Figure 4A. Among the seven metabolites, we found that 3‐(1H‐Indol‐3‐yl)‐2‐(trimethylammonio) propanoate, hippuric acid, N‐omega‐acetylhistamine, and saccharin were increased in the urine of patients with CRPs (Figure 4B,F–H), while 3‐(3‐hydroxyphenyl)‐3‐hydroxypropionic acid, N‐Methyl‐L‐proline, trimethylsilyl ester, and 3,4‐dihydroxyhydrocinnamic acid NIST were decreased in these patients (Figure 4C–E). We further explored the diagnostic value of these seven metabolites, and Figure 5 presents the corresponding ROC curves. The AUC values for 3‐(3‐hydroxyphenyl)‐3‐hydroxypropionic acid, N‐methyl‐L‐proline, trimethylsilyl ester, 3,4‐dihydroxyhydrocinnamic acid NIST, hippuric acid, saccharin, 3‐(1H‐indol‐3‐yl)‐2‐(trimethylammonio) propanoate, and N‐omega‐acetylhistamine were 0.881, 0.875, 0.859, 0.847, 0.928, 0.804, and 0.834, respectively (Figure 5A–G).

### 3.3 Demographic and Clinical Characteristics of Participants

This study involved 64 patients with CRPs and 128 healthy controls. Participants were randomly divided into training and validation sets in a 7:3 ratio. Detailed demographic characteristics are provided in Table 1. The age of participants was categorized as a binary variable using a cut‐off of 50 years [31], and metabolites were also classified into two groups based on ROC curve‐derived cut‐off values. None of the subjects had diabetes mellitus. There were no significant differences in age, sex, or metabolite levels between the two groups. Table 2 highlights the differences in age, sex, and seven metabolites between the two groups in the training set. The results indicate significant variations across all indicators, except for age.

**TABLE 1:** Baseline of clinical information and metabolite profiles for training and validation sets.

Characteristic	Group/level	Training	Validation	*p*
*n*		134	58	
Sex (%)	Male	81 (60.4)	32 (55.2)	0.601
	Female	53 (39.6)	26 (44.8)	
Age (%)	< 50	64 (47.8)	30 (51.7)	0.728
	> = 50	70 (52.2)	28 (48.3)	
Status (%)	HC	89 (66.4)	39 (67.2)	1
	CRP	45 (33.6)	19 (32.8)	
PP_0017942 (%)	Low	96 (71.6)	39 (67.2)	0.659
	High	38 (28.4)	19 (32.8)	
GC1263 (%)	Low	63 (47.0)	30 (51.7)	0.658
	High	71 (53.0)	28 (48.3)	
GC1457 (%)	Low	60 (44.8)	23 (39.7)	0.618
	High	74 (55.2)	35 (60.3)	
GC1484 (%)	Low	68 (50.7)	25 (43.1)	0.415
	High	66 (49.3)	33 (56.9)	
PN_0023236 (%)	Low	72 (53.7)	30 (51.7)	0.922
	High	62 (46.3)	28 (48.3)	
PP_0039851 (%)	Low	95 (70.9)	46 (79.3)	0.301
	High	39 (29.1)	12 (20.7)	
PN_0024153 (%)	Low	82 (61.2)	40 (69.0)	0.388
	High	52 (38.8)	18 (31.0)	

*Note:* PP_0017942, 3‐(1H‐indol‐3‐yl)‐2‐(trimethylammonio) propanoate; GC1263, 3‐(3‐hydroxyphenyl)‐3‐hydroxypropionic acid; GC1457, N‐methyl‐L‐proline, trimethylsilyl ester; GC1484, 3,4‐dihydroxyhydrocinnamic acid NIST; PN_0023236, hippuric acid; PP_0039851, N‐omega‐acetylhistamine; PN_0024153, Saccharin.

**TABLE 2:** Characteristics of healthy controls and CRP patients in the training set.

		HC	CRP	*p*
Characteristic	Group/level	(*n* = 89)	(*n* = 45)	
Sex (%)	Male	44 (49.4)	37 (82.2)	0.001
	Female	45 (50.6)	8 (17.8)	
Age (%)	< 50	48 (53.9)	16 (35.6)	0.068
	> = 50	41 (46.1)	29 (64.4)	
PP_0017942 (%)	Low	79 (88.8)	17 (37.8)	< 0.001
	High	10 (11.2)	28 (62.2)	
GC1263 (%)	Low	24 (27.0)	39 (86.7)	< 0.001
	High	65 (73.0)	6 (13.3)	
GC1457 (%)	Low	20 (22.5)	40 (88.9)	< 0.001
	High	69 (77.5)	5 (11.1)	
GC1484 (%)	Low	27 (30.3)	41 (91.1)	< 0.001
	High	62 (69.7)	4 (8.9)	
PN_0023236 (%)	Low	67 (75.3)	5 (11.1)	< 0.001
	High	22 (24.7)	40 (88.9)	
PP_0039851 (%)	Low	80 (89.9)	15 (33.3)	< 0.001
	High	9 (10.1)	30 (66.7)	
PN_0024153 (%)	Low	78 (87.6)	4 (8.9)	< 0.001
	High	11 (12.4)	41 (91.1)	

*Note:* PP_0017942, 3‐(1H‐indol‐3‐yl)‐2‐(trimethylammonio) propanoate; GC1263, 3‐(3‐hydroxyphenyl)‐3‐hydroxypropionic acid; GC1457, N‐methyl‐L‐proline, trimethylsilyl ester; GC1484, 3,4‐dihydroxyhydrocinnamic acid NIST; PN_0023236, hippuric acid; PP_0039851, N‐omega‐acetylhistamine; PN_0024153, saccharin.

### 3.4 Construction and Validation of a Predictive Model

**FIGURE 6:**
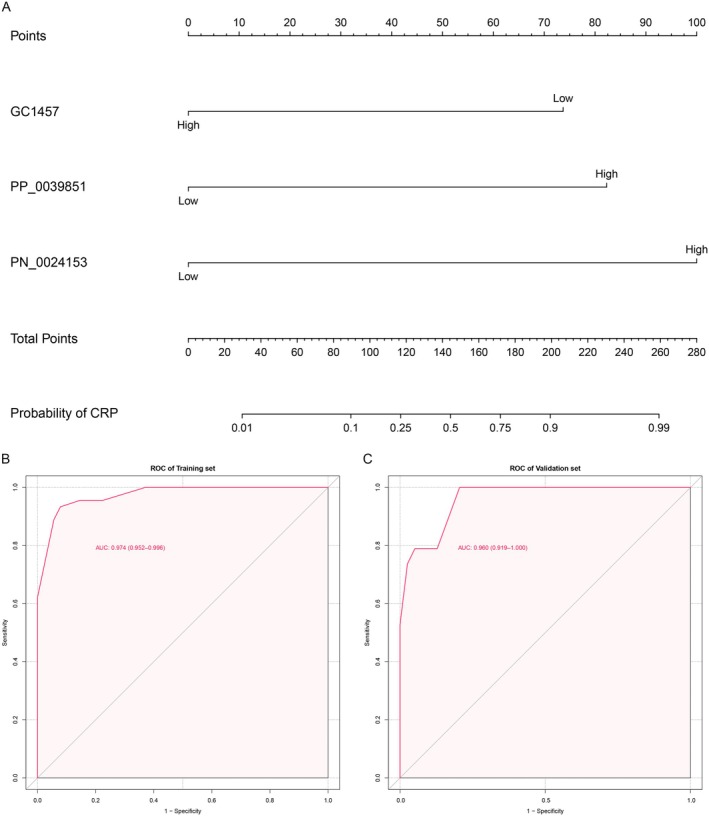
Diagnostic nomogram for CRP. (A) Diagnostic nomogram used to distinguish between patients with CRPs and healthy individuals. ROC curve of the nomogram model with the true positive rate (sensitivity) as the vertical coordinate and the false positive rate (1‐specificity) as the horizontal coordinate in the training set (B) and validation set (C). GC1457, N‐methyl‐L‐proline, trimethylsilyl ester; PP_0039851, N‐omega‐acetylhistamine; PN_0024153, saccharin.

**FIGURE 7:**
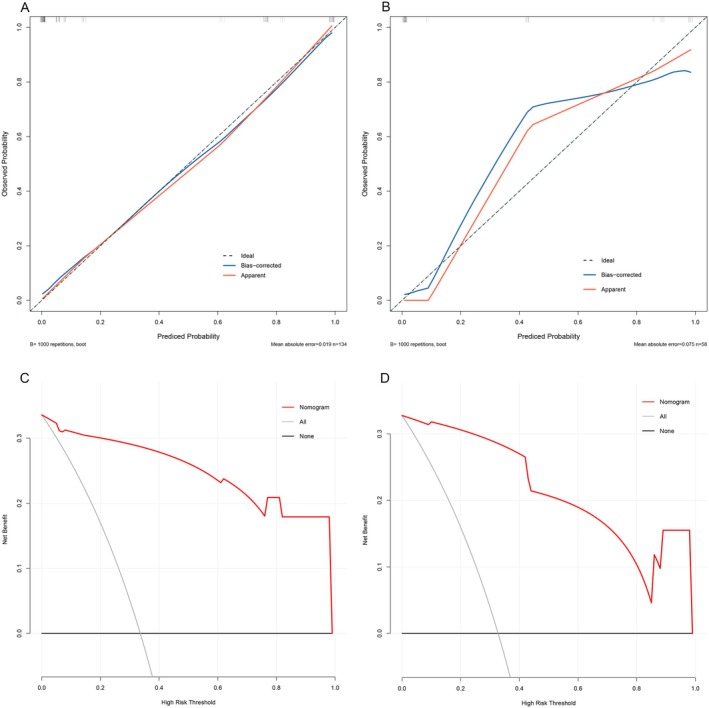
Validation of the diagnostic nomogram. Validity of the predictive performance of the nomogram in estimating CAP risk by 1000 bootstrap resampling in the training set (A) and validation set (B). DCA of the diagnostic nomogram in the training set (C) and validation set (D).

Univariate logistic regression analysis identified age, sex, 3‐(1H‐indol‐3‐yl)‐2‐(trimethylammonio) propanoate, 3‐(3‐hydroxyphenyl)‐3‐hydroxypropionic acid, N‐methyl‐L‐proline, trimethylsilyl ester, 3,4‐dihydroxyhydrocinnamic acid NIST, hippuric acid, N‐omega‐acetylhistamine, and saccharin as statistically significant factors (Table 3). Subsequent multivariate logistic regression analysis further revealed that N‐methyl‐L‐proline, trimethylsilyl ester (OR = 0.08, 95% CI: 0.01–0.8) was a protective factor against CRP, while saccharin (OR = 48.3, 95% CI: 4.44–525.32) and N‐omega‐acetylhistamine (OR = 27.91, 95% CI: 2.31–337.06) were identified as risk factors for CRP (Table 3). Additionally, although sex did not show a statistically significant difference in the multivariate logistic regression analysis (*p* = 0.059) in this study, other relevant research indicates that males have a higher prevalence of CRP [32]. To facilitate clinical application, we incorporated the three metabolites into a diagnostic nomogram for CRP (Figure 6A) and created an online tool https://gistnomo.shinyapps.io/Nomogram_for_CRP/. This nomogram represents a novel scoring system that estimates the individual probability of CRP based on metabolite levels. The model demonstrated excellent discriminatory power, with an AUC value of 0.974 in the training set (Figure 6B) and 0.960 in the validation set (Figure 6C). Calibration curves, generated through Bootstrap resampling for both the training and validation sets, indicated satisfactory calibration (Figure 7A,B). Decision curve analysis (DCA) also showed that using this nomogram provided greater clinical benefit compared to treating all or no CRP patients at various threshold probabilities (Figure 7C,D).

**TABLE 3:** Results of univariate and multivariate logistic regression models to the diagnosis of CRP.

Characteristic	Level	Univariate logistic regression		Multivariate logistic regression	
		OR (95% CI)	*p*	OR (95% CI)	*p*
Age	< 50	Reference	0.046	Reference	0.403
	> = 50	2.12 (1.01–4.44)		0.43 (0.06–3.16)	
Sex	Male	Reference	< 0.001	Reference	0.059
	Female	0.21 (0.09–0.5)		0.17 (0.03–1.07)	
GC1263	Low	Reference	< 0.001	Reference	0.849
	High	0.06 (0.02–0.15)		1.26 (0.12–13.14)	
GC1457	Low	Reference	< 0.001	Reference	0.031
	High	0.04 (0.01–0.1)		0.08 (0.01–0.8)	
GC1484	Low	Reference	< 0.001	Reference	0.342
	High	0.04 (0.01–0.13)		0.35 (0.04–3.07)	
PN_0023236	Low	Reference	< 0.001	Reference	0.649
	High	24.36 (8.55–69.42)		1.58 (0.22–11.45)	
PN_0024153	Low	Reference	< 0.001	Reference	0.001
	High	72.68 (21.78–242.57)		48.3 (4.44–525.32)	
PP_0017942	Low	Reference	< 0.001	Reference	0.291
	High	13.01 (5.33–31.75)		2.61 (0.44–15.39)	
PP_0039851	Low	Reference	< 0.001	Reference	0.009
	High	17.78 (7.04–44.92)		27.91 (2.31–337.06)	

*Note:* PP_0017942, 3‐(1H‐indol‐3‐yl)‐2‐(trimethylammonio) propanoate; GC1263, 3‐(3‐hydroxyphenyl)‐3‐hydroxypropionic acid; GC1457, N‐methyl‐L‐proline, trimethylsilyl ester; GC1484, 3,4‐dihydroxyhydrocinnamic acid NIST; PN_0023236, hippuric acid; PP_0039851, N‐omega‐acetylhistamine; PN_0024153, saccharin.

## 4 Discussion

In this study, we identified potential diagnostic biomarkers for CRPs through noninvasive urinary metabolite screening, using urine samples from 64 CRP patients and 128 controls. ROC analysis revealed high AUC values for seven metabolites, and multivariable logistic regression confirmed statistically significant variables. A nomogram was then developed to aid clinical decision‐making, incorporating four objective risk factors: sex and the metabolites N‐methyl‐L‐proline, trimethylsilyl ester, saccharin, and N‐omega‐acetylhistamine. These objective markers can assist clinicians in early CRP identification by eliminating subjectivity. Internal validation using bootstrap resampling demonstrated good discrimination and clinical applicability of the predictive model in diagnosing CRP. This study offers a novel approach to CRP detection through metabolomic analysis, which could improve diagnostic methods traditionally reliant on invasive techniques like colonoscopy.

The key advantage of this research lies in using noninvasive urine samples to detect metabolic changes linked to CRP. Urine is easily accessible and metabolite rich, making it ideal for biomarker discovery. The identified metabolites, including N‐methyl‐L‐proline, trimethylsilyl ester, saccharin, and N‐omega‐acetylhistamine, exhibit distinct metabolic profiles in CRP patients compared to healthy individuals, demonstrating their potential as biomarkers. Although the identified biomarkers are promising for CRP diagnosis, their specificity for CRP requires further study. N‐methyl‐L‐proline, a methylated derivative of proline involved in various metabolic pathways, acts as a protective factor in CRP by modulating cellular stress responses and regulating protein synthesis and tissue repair. Multivariable analysis revealed an odds ratio (OR) of 0.08 for this metabolite, indicating a strong inverse association with CRP and suggesting that elevated levels are associated with a reduced risk of polyp formation. Although N‐methyl‐L‐proline is also recognized as a biomarker of citrus fruit intake [33] and may reflect dietary habits, its significant reduction in CRP patients likely indicates specific alterations in amino acid metabolism related to CRP. The protective role of this metabolite may arise from its involvement in amino acid metabolism, which is critical for maintaining the integrity of intestinal epithelial cells. This underscores its potential as a biomarker for CRP, despite its association with dietary influences [34]. Saccharin, an artificial sweetener, was found to increase the risk of CRP (OR = 48.3). Saccharin may influence the gut microbiota and disrupt the intestinal environment, potentially promoting inflammation or dysbiosis, both known precursors to CRC [35]. Although previous studies on saccharin's carcinogenicity are inconclusive, emerging metabolomic data suggest it may impact gut health at the molecular level, making it a CRP risk factor. N‐omega‐acetylhistamine, a histamine derivative, plays a role in inflammatory responses and histamine signaling [36]. In this study, it was identified as a CRP risk factor (OR = 27.91), indicating that its elevated levels are associated with an increased likelihood of polyp formation. Histamine regulates gastrointestinal processes, including acid secretion and inflammation, which are crucial to intestinal lesion development [37, 38]. Alongside these three metabolites, four additional ones—3‐(1H‐Indol‐3‐yl)‐2‐(trimethylammonio) propanoate, 3‐(3‐hydroxyphenyl)‐3‐hydroxypropionic acid, 3,4‐dihydroxyhydrocinnamic acid NIST, and hippuric acid—were identified through machine learning, reflecting potential metabolic dysregulation linked to CRP pathogenesis. In our study, hippuric acid, although not included in the construction of the nomogram, was identified as one of the metabolites that significantly differed between healthy individuals and patients with CRP. This metabolite is known to increase with the consumption of certain foods, such as tea and fruits rich in phenolic compounds [39]. Additionally, hippuric acid has been linked to gut microbiota metabolism and has been reported as a potential biomarker for other conditions, including gastrointestinal cancers [40, 41]. While its levels can be influenced by diet, the significant elevation of hippuric acid in CRP patients suggests a potential role in CRP‐related metabolic dysregulation. Similarly, 3‐(1H‐indol‐3‐yl)‐2‐(trimethylammonio) propanoate has been identified as a potential biomarker for gastrointestinal cancers [22], indicating that it may not be specific to CRP but could also be influenced by other gastrointestinal disorders. However, its presence in CRP patients suggests that it may be associated with the early stages of CRC, highlighting its potential relevance in the progression of colorectal neoplasia. Another metabolite of interest, 3‐(3‐hydroxyphenyl)‐3‐hydroxypropionic acid, has been found to increase in urine following the consumption of polyphenol‐rich foods such as red wine and grape juice extracts [42]. Elevated levels of this metabolite have also been reported in patients with autism and schizophrenia [43, 44], and its increase has been correlated with a reduced ratio of *Mycobacterium avium* to hydroxypropionic acid in the gut [45]. Furthermore, Łukasz et al. demonstrated that 3,4‐dihydroxyhydrocinnamic acid in urine can be used to diagnose gut dysbiosis [46]. In our study, both 3‐(3‐hydroxyphenyl)‐3‐hydroxypropionic acid and 3,4‐dihydroxyhydrocinnamic acid were found to be reduced in the urine of CRP patients compared to healthy controls. Whether these metabolites influence the development of CRP by modulating the gut microbiota requires further investigation. To further validate the specificity of these biomarkers, future studies should include longitudinal analyses to determine whether these metabolites can predict the progression of benign polyps to malignant lesions. Such studies would help clarify their role in early detection and prevention strategies. Additionally, detailed dietary and lifestyle assessments should be incorporated to better understand the impact of external factors on metabolite levels. Finally, validation of these biomarkers in larger, multicenter cohorts is essential to confirm their diagnostic accuracy across diverse populations and settings.

Previous studies underscore the utility of urinary metabolomics in diagnosing various diseases, including cancer, supporting the feasibility of using these biomarkers in routine clinical practice [47]. For example, Mona et al. demonstrated that urine metabolomics offers comprehensive metabolic information for biomarker discovery [17], while Joanna et al. highlighted its potential in cancer diagnostics [48]. Although urine is an excellent metabolic matrix due to its noninvasive collection and rich metabolic information, future studies could benefit from integrating other biological matrices, such as blood, tissue, or stool samples [49]. Blood metabolomics could provide additional insights into systemic metabolic changes associated with CRP, while tissue metabolomics could reveal localized alterations in the colorectal epithelium. Integrating data from multiple matrices could enhance the robustness and accuracy of biomarker identification, offering a more comprehensive understanding of the metabolic pathways involved in CRP development. Furthermore, longitudinal studies incorporating multiple matrices could help elucidate the temporal dynamics of metabolic changes during the progression from benign polyps to malignant lesions, thereby improving early detection and prevention strategies.

The nomogram based on these biomarkers holds significant translational value, offering clinicians a practical tool for estimating individual CRP risk, allowing for personalized screening and early intervention strategies. The high AUC values in both the training and validation sets demonstrate the model's robustness and reliability.

However, several limitations need to be addressed. The relatively small sample size, particularly in the validation cohort, calls for larger multicenter studies to confirm the diagnostic accuracy of these urinary biomarkers and the nomogram's predictive power across diverse populations. Additionally, while the identified metabolites show promise, their biological roles in CRP progression are still unclear. Further functional studies are needed to elucidate the underlying mechanisms and validate these biomarkers in other cohorts. Another challenge is the lack of longitudinal data. The current cross‐sectional design limits our ability to determine whether the identified metabolites are causally linked to CRP development or merely reflect existing disease. Future studies should focus on longitudinal analyses to assess whether these biomarkers can predict the progression of benign polyps to malignant lesions, which is crucial for improving early detection and prevention strategies.

## 5 Conclusion

This study lays a solid foundation for using urinary metabolites to diagnose CRPs and provides a valuable tool for clinical risk assessment through the development of a nomogram. While further research is needed to validate these findings, incorporating metabolomic biomarkers into routine screening has the potential to revolutionize CRP diagnosis and improve patient outcomes.

## Author Contributions

J.L., Q.T., and Y.W. recruited study subjects. Y.X., Y.J., and Z.L. procured and collated patients' clinical data. Metanotitia Inc. performed laboratory work, including sample analysis and testing. Y.X., C.Z., and Y.C. analyzed and interpreted the data and wrote the manuscript. Y.C. and C.Z. designed and supervised the study.

## Ethics Statement

This study was approved by the Ethics Committee of the First Affiliated Hospital of Nanchang University (Ethics No. ChiCTR1900022425). All patients had signed the written informed consent.

## Conflicts of Interest

The authors declare no conflicts of interest.

## Supporting information


**Figure S1.** Relative peak intensity of metabolites in the training set. (A) PP_0017942, 3‐(1H‐indol‐3‐yl)‐2‐(trimethylammonio) propanoate; (B) GC1263, 3‐(3‐hydroxyphenyl)‐3‐hydroxypropionic acid; (C) GC1457, N‐Methyl‐L‐proline, trimethylsilyl ester; (D) GC1484, 3,4‐dihydroxyhydrocinnamic acid NIST; (E) PN_0023236, hippuric acid; (F) PP_0039851, N‐omega‐acetylhistamine; and (G) PN_0024153, saccharin.

## Data Availability

The data that support the findings of this study are available from the corresponding author upon reasonable request.
