# Supplementary figures and images for: Identification of Diagnostic Biomarkers for Colorectal Polyps Based on Noninvasive Urinary Metabolite Screening and Construction of a Nomogram
Source: Cancer Med. 2025 Apr 8;14(7):e70762. doi: 10.1002/cam4.70762 (PMC11978731; doi:10.1002/cam4.70762)

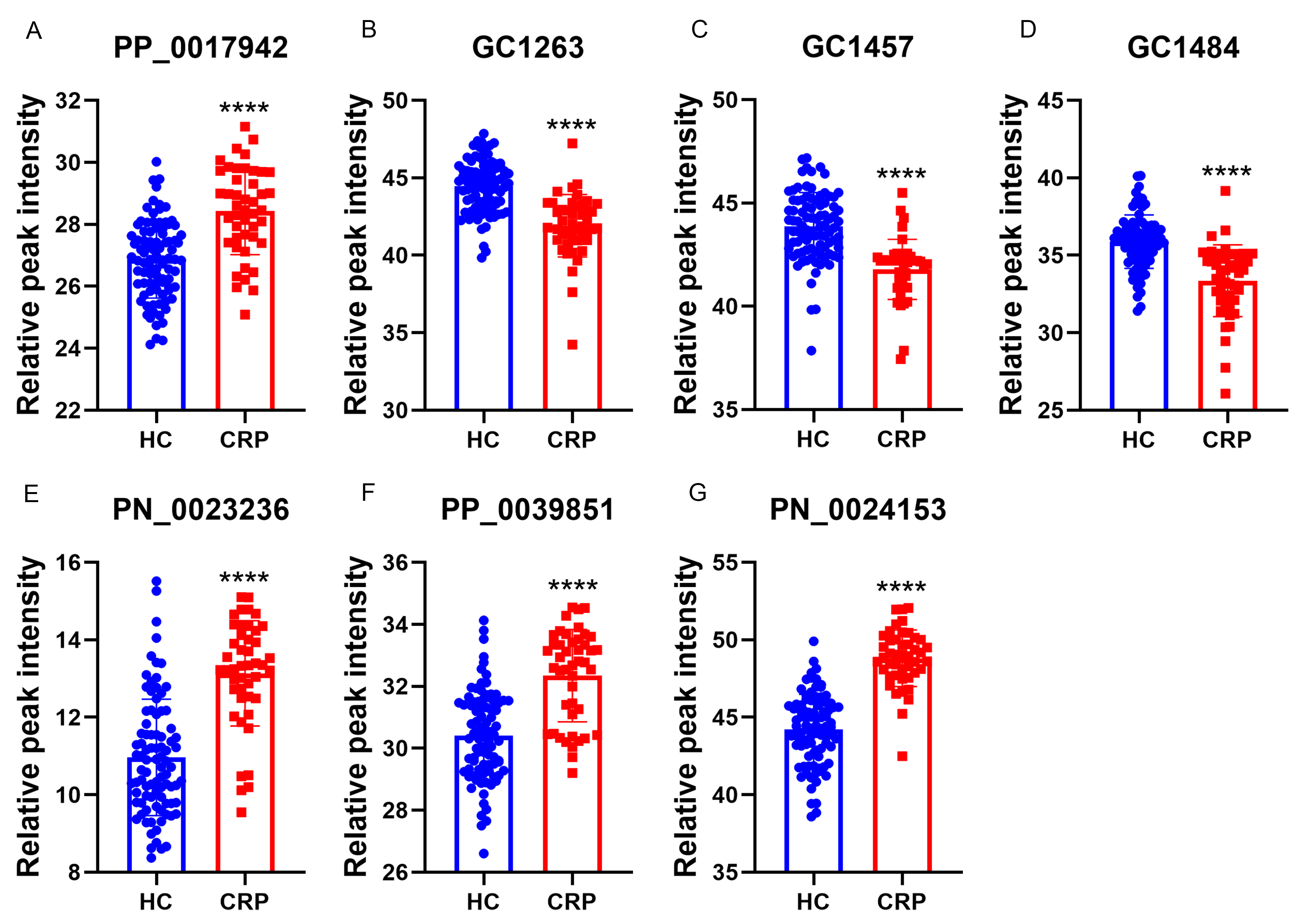

Supplement: Supplementary file 1 — Figure S1. Relative peak intensity of metabolites in the training set. (A) PP_0017942, 3‐(1H‐indol‐3‐yl)‐2‐(trimethylammonio) propanoate; (B) GC1263, 3‐(3‐hydroxyphenyl)‐3‐hydroxypropionic acid; (C) GC1457, N‐Methyl‐L‐proline, trimethylsilyl ester; (D) GC1484, 3,4‐dihydroxyhydrocinnamic acid NIST; (E) PN_0023236, hippuric acid; (F) PP_0039851, N‐omega‐acetylhistamine; and (G) PN_0024153, saccharin. [file CAM4-14-e70762-s001.tif]
